# Identification of Feline Interferon Regulatory Factor 1 as an Efficient Antiviral Factor against the Replication of Feline Calicivirus and Other Feline Viruses

**DOI:** 10.1155/2018/2739830

**Published:** 2018-06-12

**Authors:** Yongxiang Liu, Xiaoxiao Liu, Hongtao Kang, Xiaoliang Hu, Jiasen Liu, Jin Tian, Liandong Qu

**Affiliations:** Division of Zoonosis of Natural Foci, State Key Laboratory of Veterinary Biotechnology, Harbin Veterinary Research Institute, Chinese Academy of Agricultural Sciences, Harbin 150001, China

## Abstract

Interferons (IFNs) can inhibit most, if not all, viral infections by eliciting the transcription of hundreds of interferon-stimulated genes (ISGs). Feline calicivirus (FCV) is a highly contagious pathogen of cats and a surrogate for Norwalk virus. Interferon efficiently inhibits the replication of FCV, but the mechanism of the antiviral activity is poorly understood. Here, we evaluated the anti-FCV activity of ten ISGs, whose antiviral activities were previously reported. The results showed that interferon regulatory factor 1 (IRF1) can significantly inhibit the replication of FCV, whereas the other ISGs tested in this study failed. Further, we found that IRF1 was localized in the nucleus and efficiently activated IFN-*β* and the ISRE promoter. IRF1 can trigger the production of endogenous interferon and the expression of ISGs, suggesting that IRF1 can positively regulate IFN signalling. Importantly, the mRNA and protein levels of IRF1 were reduced upon FCV infection, which may be a new strategy for FCV to evade the innate immune system. Finally, the antiviral activity of IRF1 against feline panleukopenia virus, feline herpesvirus, and feline infectious peritonitis virus was demonstrated. These data indicate that feline IRF1 plays an important role in regulating the host type I IFN response and inhibiting feline viral infections.

## 1. Introduction

 Feline calicivirus (FCV) is a highly contagious pathogen of cats and usually causes mild to serious oral and upper respiratory tract disease [1]. FCV belongs to Vesivirus, a genus of Caliciviridae, which comprises small RNA viruses of both medical and veterinary importance [2]. The human noroviruses (HuNoV) and many other caliciviruses are difficult to cultivate in vitro, and the lack of in vitro infection models and robust small-animal models has posed barriers to the development of virus-specific therapies and preventive vaccines [3]. Although recent studies indicated that limited HuNoV replication can occur in immortalised B cells [4, 5], this may not be a perfect cellular model for research about the characteristics of HuNoV. As FCV can replicate well and produce a significant cytopathic effect (CPE) in vitro, it has been widely used by researchers as a surrogate for Norwalk virus (NV) [6].

Only recently was the Felis catus genome sequenced, so little is known about feline innate immunity and the interaction of FCV with the feline innate immunity, which limits the understanding of the pathogenesis of FCV and other feline viruses [7]. The nonstructural protein p39 of the FCV F4 strain could suppress the production of IFN-*β* by preventing IRF3 activation [8]. Meanwhile, our earlier study found that infection of FCV-2280 led to a robust release of IFN-*β*, but other FCV strains failed [7]. In addition, the FCV strain 2280 proteinase-polymerase (PP) protein suppresses luciferase reporter gene expression driven by endogenous and exogenous promoters, which contributes to the inhibition of host cell transcription [6].

FCV is very sensitive to interferons, and IFN-*β* can suppress the infection of FCV. Treatment with these IFNs reduced the viral yield of FCV F9 [9, 10]. Feline IFN-*ω* has been marketed in Japan (Toray Industries, Tokyo, Japan) and is used to treat feline calicivirus and canine parvovirus infections [11, 12]. We have reported that the feline IFN-*β* can efficiently inhibit the replication of the FCV 2280 strain. Interferons (IFNs) are a component of an early response to invading pathogens and induce the expression of hundreds of IFN-stimulated genes (ISGs) [13]. The mechanisms of the antiviral action of some ISGs have been identified. One of the key enzymes involved in the function of interferons (IFNs), 2′-5′oligoadenylate-dependent ribonuclease L (RNase L), can degrade single-stranded viral RNAs [14]. Interferon-induced protein with tetratricopeptide repeats 3 (IFIT3) inhibits PRRSV replication in MARC-145 cells by mediating the dsRNA-induced production of IFN-*β* [15]. ISG20 is an interferon-inducible 3′-5′ exonuclease that inhibits the replication of several human and animal positive-strand RNA viruses, such as hepatitis A virus, hepatitis C virus, bovine viral diarrhoea virus, and yellow fever virus [16]. Interferon-stimulated gene 15 (ISG15) is a ubiquitin-like protein strongly induced by type I IFN and functions as both an antiviral and an immunoregulatory molecule. Interferon-induced GTP-binding protein Mx1 can inhibit different members of Rhabdoviridae, Paramyxoviridae, Orthomyxoviridae, and Bunyaviridae as well as dsDNA viruses, including hepatitis B virus (HBV) and African swine fever virus (ASFV) [17]. SAMHD1 restricts herpes simplex virus 1 in macrophages by limiting DNA replication [18] and restricts the reverse transcription of HIV-1 in quiescent CD4+ T-cells [19]. In addition, HIV is sensitive to many ISGs, such as APOBEC3G, TRIM5*α*, Tetherin, and MX2 [20].

While FCV is sensitive to interferons, the ISGs that could inhibit the replication of feline calicivirus have not been identified. Here, we evaluated the antiviral activity of ten feline ISGs (IRF1, RNase L, SAMHD, ISG15, ISG20, IFIT3, MX-1, GBP6, OASL, and IRF2) against FCV using a transient transfection method and found that feline IRF1 could efficiently suppress the replication of FCV. Further, we found that feline IRF1 can trigger the production of endogenous interferon and the expression of ISGs, suggesting that IRF1 can positively regulate IFN signalling. Importantly, the mRNA and protein levels of IRF1 were reduced upon FCV infection, which may be a new strategy for FCV to evade the innate immune system. Finally, the antiviral activity of IRF1 against feline panleukopenia virus, feline herpesvirus, and feline infectious peritonitis virus was also demonstrated.

## 2. Materials and Methods

### 2.1. Viruses and Cells

Crandell Rees feline kidney (CRFK) cells were maintained in Dulbecco's modified minimum essential medium (DMEM) (Gibco) containing 10% foetal bovine serum (FBS, Gibco), 100 U/ml penicillin, and 100 *μ*g/ml streptomycin. FCV strain F9, FHV-1 strain HR-1, FPV strain VR-638, and FIPV strain 2034 were propagated and titred in CRFK cells.

### 2.2. Plasmids Construction

The p3×Flag-IRF1, p3×Flag-RNase L, p3×Flag-SAMHD, p3×Flag-ISG15, p3×FlagISG20, p3×Flag-IFIT3, p3×Flag-MX-1, p3×Flag-GBP6, p3×Flag-OASL and p3×Flag-IRF2 plasmids express Flag-tagged feline IRF1 (Accession number: XM_003980752.4), RNase L (Accession number: XM_006942762.2), SAMHD (Accession number: XM_003983547.3), ISG15 (Accession number: NM_001130843.2), ISG20 (Accession number: XM_019831901.1), IFIT3 (Accession number: XM_011287196.2), MX-1 (Accession number: XM_006935851.2), GBP6 (Accession number: XM_003990288.3), OASL (Accession number: XM_003994734.3), and IRF2 (Accession number: XM_019828250.1), respectively. The pMyc-IRF1 plasmid expresses IRF1 with a Myc-tag. The plasmid p3×Flag-IFN-*β* encodes the Flag-tagged feline IFN-*β* and the plasmid pIFN-*β*-Luc contains a luciferase (Luc) expression cassette driven by the feline IFN-*β* promoter as described previously [7]. The plasmid pISRE-TA-Luc expressed the luciferase reporter gene being under control of the interferon stimulation response element (ISRE). The pRL-TK plasmid (Promega, Madison, WI, USA) that encodes Renilla luciferase was used as an internal control for the normalization of gene transfection efficiency. The pEGFP-IRF1 plasmid expresses the feline IRF1 with an EGFP tag. The primers for construction of IRF1 are shown in Table 1.

### 2.3. Luciferase Test

The protocol for this luciferase assay has been described previously [7]. Briefly, CRFK cells (5×10^4^/well) grown in 48-well plates were cotransfected with 0.5 *μ*g/well reporter plasmid, 0.02 *μ*g/well plasmid pRL-TK (Promega) (as an internal control for normalization of the assay system), or the indicated expression plasmid. Cells were infected with SeV (100 haemagglutinating activity units/well) at 12 h after cotransfection. The cells were lysed at 8-10 h postinfection, and the firefly and Renilla luciferase activities were measured with the dual-luciferase reporter assay system (Promega), according to the manufacturer's protocol. Relative luciferase activity in each sample was determined using the ratio between the activities of firefly and Renilla luciferases.

### 2.4. Evaluation of the Antiviral Activity of Feline ISGS

Briefly, CRFK cells (2×10^5^/well) were seeded into 24-well culture plates for 24 h and then were transfected with 1 *μ*g of ISG expression plasmids or mock-transfected with 1 *μ*g of p3×Flag-CMV-10. FCV strain F9 with an MOI of 0.01 was inoculated into cells at 24 h after transfection. The cell supernatant and cells were harvested for the examination of viral titres and viral RNA, respectively, at 24 h postinfection.

### 2.5. Quantitative Real-Time RT-PCR Assay

The protocol for quantitative real-time RT-PCR has been described previously [21]. The cDNA was prepared with the AMV-reverse transcription kit (Takara, Japan), according to the manufacturer protocol. Real-time quantitative PCR targeting the FCV gene and IRF1 gene was carried out using an Agilent Mx3005P according to the manufacturer instructions. The relative mRNA expression levels were calculated by the 2-ΔΔCT method using GADPH as an internal control for normalization. The primers are shown in Table 1.

### 2.6. Viral Titre Test

The protocol for viral titration has been described in our previous study [1]. Briefly, tenfold diluted virus stocks were prepared with DMEM without serum, and 0.1 ml of each dilution was inoculated into cells seeded into a 96-well culture plate. After 1 h of adsorption, the supernatant was discarded and 0.1 ml fresh DMEM containing 1% FBS and 1% penicillin-streptomycin was added to each well. The CPE was observed at 72 h postinoculation and viral titres were expressed as the median tissue culture infectious dose (TCID50)/mL according to the method of Reed and Müench [22].

For FPV infection experiment, the viral yields were determined via direct fluorescence assay (DFA) using FITC-conjugated anti-canine parvovirus monoclonal antibody (CJ-F-CPV-MAB, VMRD).

### 2.7. Western Blot Analysis

Cells were washed with cold PBS and lysed with RIPA Lysis Buffer (Beyotime Institute of Biotechnology, Nantong, China) with 0.1 mM PMSF, and then lysates were cleared by centrifugation at 12,000 g for 5 min at 4°C. Equal amounts of protein samples were separated by 10% SDS-PAGE and transferred onto nitrocellulose membranes (Millipore). The membranes were blocked with 5% skim milk for 1-2 h at room temperature and then incubated for 1 hour at room temperature with mouse anti-FLAG M2 MAb (1804, Sigma), rabbit anti-Myc polyclonal antibody (ab9106, Abcam), rabbit anti-IRF1 monoclonal antibody (ab186384, Abcam), rabbit anti-GADPH antibody (ab22555, Abcam), and mouse anti-FCV VP1 monoclonal antibody (made by our laboratory).

After three rinses in TBST buffer, the membranes were incubated at room temperature for 1 h with IRDye 800DX conjugated anti-rabbit IgG or IRDye 800-conjugated anti-mouse IgG (1:8000; Rockland Immunochemicals) diluted with TBST as a secondary antibody. After the third wash, membranes were visualized and analysed with an Odyssey infrared imaging system (LI-COR Biosciences).

### 2.8. Statistics

The significant differences between the experimental groups were determined with the paired* t*-test and one-way ANOVA with Prism 5.0 software (GraphPad Software). A p value of <0.05 was chosen to indicate significance.

## 3. Results

### 3.1. Screening of Feline ISGs That Could Inhibit the FCV Replication

**Figure 1:**
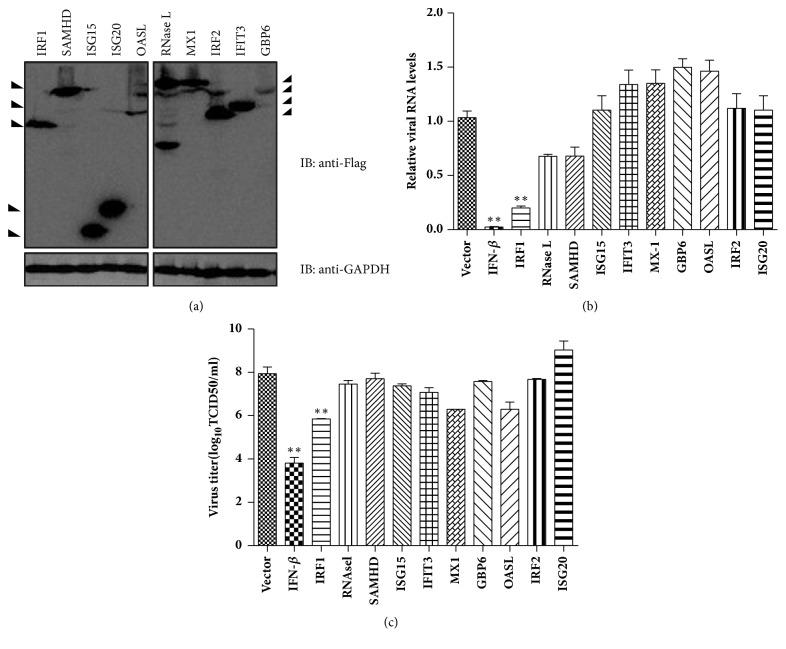
Identification of feline ISGs that inhibit FCV infection. (a) CRFK cells were transfected with a plasmid expressing 10 different ISGs: IRF1, RNase L, SAMHD, ISG15, ISG20, IFIT3, MX-1, GBP6, OASL, and IRF2. At 24 h posttransfection, their expression levels were determined by Western blot using an anti-Flag antibody. (b, c) Effect of ISGs expression on FCV replication. CRFK cells were transfected with 500 ng/well of the ISG plasmid or empty vector or IFN-*β* expression plasmid (positive control). At 24 h posttransfection, cells were infected with FCV F9 at an MOI of 0.01. At 24 h posttransfection, cellular total RNA was extracted and the relative levels of FCV genome were tested with qRT-PCR (b). The supernatants were collected for the detection of viral titres (c). Error bars represent standard deviations and each sample was run in triplicate.*∗*: P<0.05; *∗∗*: P<0.01.

To explore which feline ISGs could inhibit the replication of FCV, ten feline ISGs with antiviral activities that have been described were cloned into the p3×Flag-CMV10 vector. The expression of these feline ISGs with a Flag tag was identified by Western blot using anti-Flag antibody (Figure 1(a)). Next, CRFK cells were transfected with an empty vector or ISG expression plasmid for 24 h, and then FCV strain F9 was inoculated into cells for another 24 h. We first analysed the viral RNA levels between the vector and ISG transfection groups. The expression of feline IFN-*β* (positive control), IRF1, RNase L, and SAMHD significantly reduced viral RNA levels compared with those of the vector transfection group (Figure 1(b)). Among these ISGs, IRF1 was the most efficient inhibitor and decreased viral RNA production by at least 80% (Figure 1(b)). The result of viral titre analysis also showed that the expression of IRF1 significantly inhibited viral yield, but the expression of RNase L and SAMHD did not affect viral titres in cellular supernatants (Figure 1(c)). These data showed that feline IRF1 (fe-IRF1) is a potent inhibitor for FCV.

### 3.2. Overexpression of Fe-IRF1 Inhibits the Replication of FCV in a Dose-Dependent Manner

**Figure 2:**
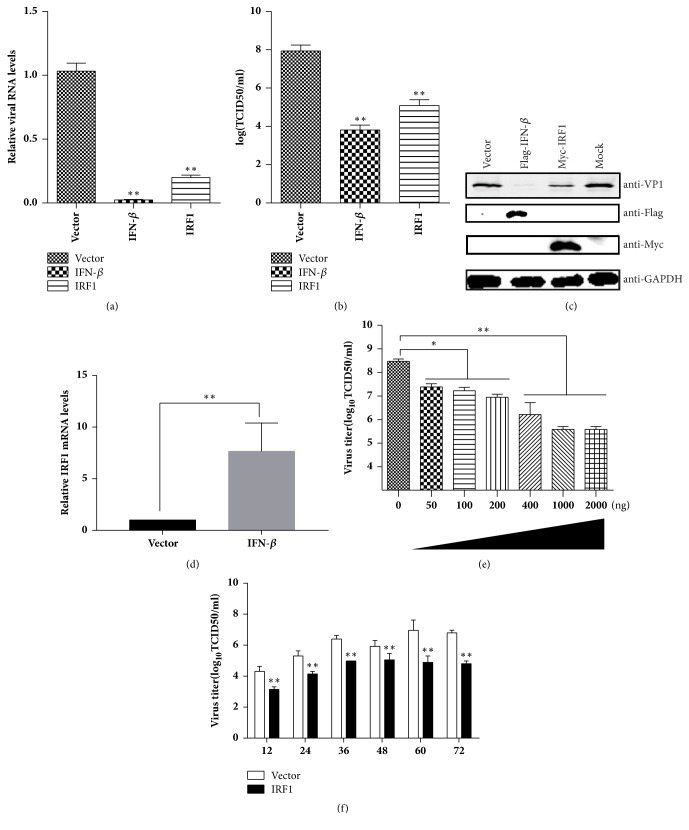
Fe-IRF1 inhibits FCV replication. (a, b, c) CRFK cells were transfected with 500 ng/well of the pMyc-IRF1. At 24 h posttransfection, cells were infected with FCV F9 at an MOI of 0.01 for 24 h. The relative genome RNA (a), viral yields (b), and the expression of VP1 (c) of FCV were detected. (d) CRFK cells were transfected with 500 ng/well of the p3×Flag-IFN-*β* or p3×Flag-CMV-10. The mRNA levels of endogenous IRF1 were detected by qRT-PCR 24 hours posttransfection. (e) p3×Flag-IRF1 (50, 100, 200, 400, 1000, and 2000 ng) were transfected into cells. At 24 h posttransfection, cells were infected with FCV F9 at an MOI of 0.01. At 24 h posttransfection, the viral yields of each sample were determined. (f) The cells were transfected with 500 ng/well of the pMyc-IRF1 plasmid or empty vector for 24 h; then FCV with an MOI of 0.01 was inoculated. At 12 h, 24 h, 36 h, 48 h, 60 h, and 72 h postinoculation, the viral yields of each sample were determined. Error bars represent standard deviations and each sample was run in triplicate.*∗*: P<0.05; *∗∗*: P<0.01.

To exclude the effect of vector on the replication of FCV, fe-IRF1 was cloned into another eukaryotic expression plasmid pCMV-Myc named pMyc-IRF1. CRFK cells were transfected with pMyc-IRF1 or an empty vector. After 24 h of transfection, the cells were inoculated with FCV F9 at an MOI of 0.01 for 24 h. The levels of viral RNA (Figure 2(a)) and titres (Figure 2(b)) were analysed. Indeed, both levels were significantly suppressed, and the decreased expression of FCV capsid protein VP1 by fe-IRF1 was also detected (Figure 2(c)). Besides, IRF1 is an important ISG and transfected Fe-IFN-*β* plasmid can significantly increase the expression of fe-IRF1 mRNA with 7.5-fold (Figure 2(d)). Moreover the inhibitory effect of IRF1 occurred in a dose-dependent manner (Figure 2(e)).

Next, we compared the growth curve of FCV in the control cells and fe-IRF1 transfected cells. Compared with the mock-transfected cells, viral yield in the fe-IRF1 transfected cells was significantly lower than that in mock-transfected cells at each time point (Figure 2(f)). At 60 h postinfection, the expression of fe-IRF1 resulted in an approximately 2 log10TCID50/ml reduction (Figure 2(f)).

Based on these results, we concluded that fe-IRF1 is an efficient antagonist of FCV.

### 3.3. Fe-IRF1 Shares A Conserved DNA-Binding Domain

**Figure 3:**
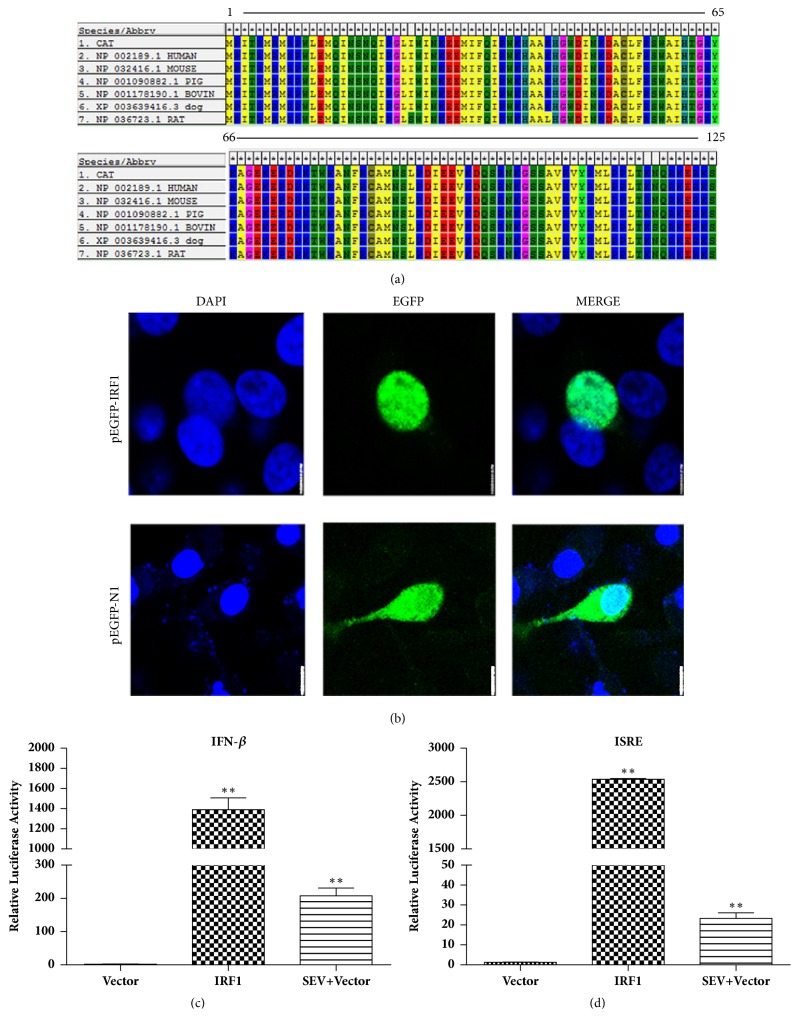
Fe-IRF1 is located in the nucleus and can activate the IFN-*β* pathway. (a) The N terminus sequences from 1 to 125 amino acid of human, mouse, rat, pig, bovine, dog, and cat IRF1 were aligned using MEGA5. (b) CRFK cells were transfected with pEGFP-IRF1 and subjected to confocal microscopy. (c, d) CRFK cells were cotransfected with 0.25 *μ*g/well of the reporter plasmid pIFN-*β*-Luc (c) or pISRE-TA-Luc (d) and 0.25 *μ*g/well of p3×Flag-IRF1 or empty vector, along with 0.02 *μ*g /well of pRL-TK plasmid. Luciferase assays were performed at 24 h posttransfection. The SeV infection group serves as a positive control. The data shown represent the mean ± SD for three independent experiments. *∗*: P<0.05; *∗∗*: P<0.01.

IRF-1 is a transcriptional regulation factor, and its N-terminal 125 amino acids (N-125) encoding the DNA-binding domain (DBD) are structurally and functionally conserved among the IRF family of proteins [23]. We analysed the N-terminal 125 amino acid sequences of IRF1 from human, mouse, pig, bovine, dog, rat, and cat using MEGA5.0. As shown in Figure 3(a), the N-125 of fe-IRF1 shares 100% similarity with that of human, pig, and dog, suggesting that the N-125 of fe-IRF1 is conserved.

To explore the subcellular localization of fe-IRF1, the fe-IRF1 CDS were inserted into pEGFP-N1 and named pEGFP-IRF1. Confocal microscopy indicated that the fe-IRF1 fusion protein was localized in the nucleus, which is the same subcellular localization as other IRF1s.

### 3.4. Overexpression of Fe-IRF1 Activates IFN-*β* and ISRE Promoters

As an important signalling transcriptional regulation factor, IRF1 plays a pivotal role in the activation of type I IFN responses during infections with viruses and bacteria and due to other responses [24]. To examine whether fe-IRF1 activates the IFN-*β* signalling pathway, CRFK cells were cotransfected with the fe-IRF1 plasmid and IFN-*β* (Figure 3(c)) or the ISRE (Figure 3(d)) promoter reporter. The results revealed that fe-IRF1 overexpression significantly enhanced luciferase activities relative to that of empty vector-transfected cells, suggesting that fe-IRF1 activated IFN-*β* and the ISRE promoter. In fact, the transfected fe-IRF1 can significantly upregulate the mRNA expression of ISG15 (500-fold), IFITM1 (40-fold), and Viperin (300-fold) (Figure.  S1).

The results indicated that fe-IRF1 is a positive feedback factor in the type I IFN response, triggering the IFN-*β* and downstream signalling pathway.

### 3.5. FCV Infection Decreases the Expression of Fe-IRF1

**Figure 4:**
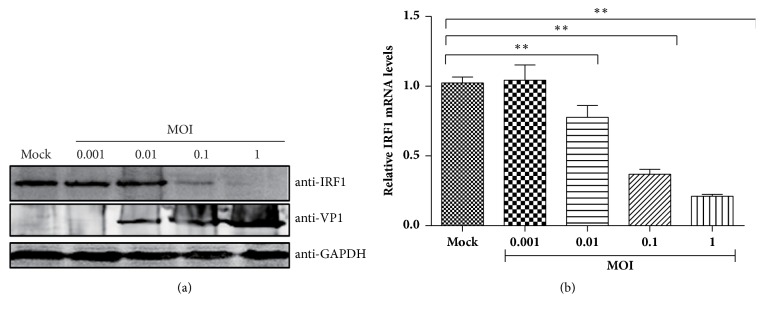
Expression of IRF1 is downregulated by FCV infection. CRFK cells were inoculated with different MOIs as indicated. Twenty-four hours later, the expression of IRF1 was identified by Western blot using an anti-IRF1 antibody (a). The relative mRNA levels of IRF1 were assessed using qRT-PCR (b). The data shown represent the mean ± SD for three independent experiments. *∗*: P<0.05; *∗∗*: P<0.01.

IRF1 can be induced by viral infection to eliminate viral infection [23]. Since overexpression of fe-IRF1 can inhibit the proliferation of FCV F9, we wanted to know whether infection with FCV can induce the expression of IRF1. To identify if the expression of IRF1 was increased upon FCV infection, cells were infected with different MOIs ranging from 0.001 to 1, and then the protein levels of cellular IRF1 and FCV VP1 were evaluated at 24 h postinfection. The result demonstrated that the protein level of the endogenous fe-IRF1 was reduced by viral infection in a viral dose-dependent manner (Figure 4(a)).

Then, to explore whether FCV infection also reduces fe-IRF1 mRNA or not, the mRNA levels of fe-IRF1 were tested by quantitative real-time RT-PCR assay. We found that the mRNA of IRF1 was also downregulated with the infection of FCV (Figure 4(b)). These results indicated that FCV infection led to decreased levels of both fe-IRF1 protein and mRNA.

### 3.6. Fe-IRF1 Can Also Inhibit Other Feline Viruses

**Figure 5:**
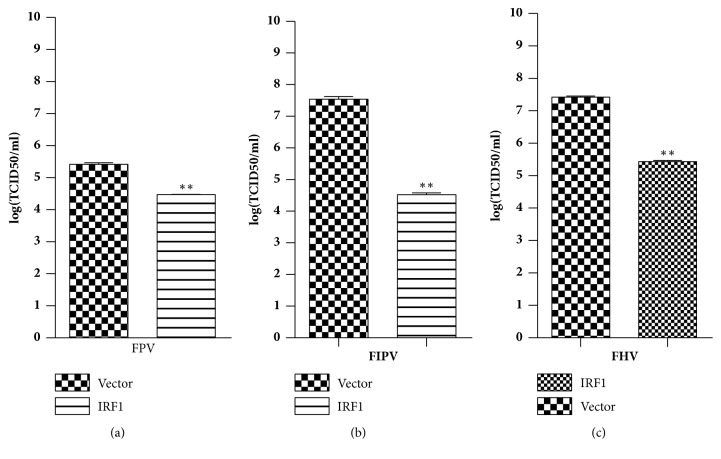
Fe-IRF1 can suppress other feline viral pathogens. CRFK cells were transfected with p3×Flag-IRF1 or empty vector for 24 h. Then, the cells were inoculated with an MOI of 0.01 of FPV (a), FIPV (b), and FHV (c). The supernatants were collected at 24 h postinoculation. The viral yields were presented as TCID50. The data shown represent the mean ± SD for three independent experiments. *∗∗*: P<0.01.

To examine whether fe-IRF1 can also inhibit the replication of other feline viruses, the fe-IRF1-transfected F81 cells were inoculated with an MOI of 0.01 feline herpes virus (FHV), feline infectious peritonitis virus (FIPV), and feline panleukopenia virus (FPV). At 48 h postinoculation, the viral yields were tested. The results indicated that overexpression of fe-IRF1 can efficiently impede the replication of FPV (Figure 5(a)), FIPV (Figure 5(b)), and FHV (Figure 5(c)). The viral yields were reduced approximately 1, 2.2, and 2 log (TCID50/mL), respectively, suggesting that fe-IRF1 has a broad-spectrum antiviral activity.

## 4. Discussion

The IFN system has a profound role in inducing the expression of antiviral proteins encoded by interferon-stimulated genes [25]. The products of these ISGs exert numerous antiviral effector functions, many of which are still not fully described [26]. In this study, ten feline ISGs were cloned and successfully expressed, and through a screening assay, we found that the ectopic expression of only fe-IRF1 could significantly inhibit the replication of FCV. IRF1 is not only an important ISG but also a key transcriptional regulator factor [27] in the transcriptional regulation of the IFN-*β* gene [23]. More importantly, infection of FCV can suppress the endogenous expression of fe-IRF1, suggesting a new strategy for FCV to evade the host antiviral response.

IRF1 is a transcription factor that regulates the innate and adaptive immune responses [28]. The IRF family comprises transcription factors that regulate the expression of interferon (IFN) and IFN-stimulated genes (ISGs) by binding to elements in their promoters [29, 30]. IRF1 was the first IRF family member known to activate the IFN-*β* promoter and found to be constitutively expressed at a low basal level in most cell types [31]. The identified mammal IRF1 is constitutively localized in the nucleus [32, 33], but some fish homologues of IRF1 are not strictly localized to the nucleus [34].

The murine IRF1 contains two nuclear localization signal sequences (NLS), 120RKERKSK and 132KSKTKRK. A 24-amino acid sequence that contains both sequences was found to mediate nuclear translocation of IRF1 [35]. The N-terminal 125 amino acids encoding the DNA-binding domain (DBD) are structurally and functionally conserved among the IRF family of proteins [23]. We found that fe-IRF1 has the signal sequences for a NLS and its DBD domain, which were also conserved. The consensus binding motif of IRF1 (5′-G(A)AAA G/C T/C GAAA G/C T/C-3′[36]) appears within upstream of several IFN-stimulated genes (ISGs) and can strongly activate the IFN-*β* and ISRE promoter by its DBD domain, which can upregulate the expression of many genes such as IFN-*α*/*β* and many ISG genes such as 2′, 5′-OAS, protein kinase R[23], and Viperin [37]. It is easy to understand why IRF1 can efficiently inhibit the replication of many viruses.

IRF1 mRNA rises in response to IFNs, double-stranded RNA (dsRNA), cytokines, some hormones [23], and viral infection [38] and then promotes the expression of antiviral proteins and restricts the replication of many viruses [28, 38, 39]. However, to subvert innate immunity, many viruses have evolved the strategy to suppress the expression of IRF1 [40]. In this study, we found that FCV infection resulted in decreased levels of both fe-IRF1 protein and mRNA, which was not consistent with the previous report that porcine IRF1 was continuously increased in the TGEV-infected cells [38]. Members of many different viral families inhibit the expression of host genes during the process of viral replication by affecting mRNA transcription, processing and transport, and translation [41]. Calicivirus has also developed diverse strategies to subvert or regulate the host protein synthesis machinery to their advantage [42]. FCV strain F9 was shown to shut off host protein synthesis by the cleavage of the eukaryotic translation initiation factors eIF4GI and eIF4GII [43], which may be one of the factors for the reduced fe-IRF1 expression. However, no report describes FCV infection-mediated downregulation of host gene mRNA, which is a novel strategy for FCV to inhibit host antiviral response. The precise mechanism remains to be further investigated.

In conclusion, we demonstrate for the first time that fe-IRF1 inhibits the replication of FCV and other feline viruses. We have also provided evidence that FCV infection suppresses the expression of IRF1 by reducing the level of fe-IRF1 mRNA, which highlights a potential immune evasion strategy for FCV.

## Figures and Tables

**Table 1:** Primers used in this study.

Primer	Sequence (5′–3′)	Usage
Flag-IRF1-F	attgcggccgcgATGCCCATCACTCGGATGCGCATGAGA	Amplification of IRF1
Flag-IRF1-R	atttctagaCTACGGTGCACAAGGAATGGCCTGGAT	
Myc- IRF1-F	attctcgaggtATGCCCATCACTCGGATGCG	
Myc- IRF1-R	attgcggccgcCTACGGTGCACAAGGAATGG	
GFP- IRF1-F	attctcgagATGCCCATCACTCGGATGCGC	
GFP- IRF1-R	attgaattcgCGGTGCACAAGGAATGGCCT	
Q-GAPDH-F	TGACCACAGTCCATGCCATC	qRT-PCR for detection of GAPDH
Q-GAPDH-R	GCCAGTGAGCTTCCCGTTCA	
Q-FCV-F	ATGATTTGGGGTTGTGATGT	qRT-PCR for detection of FCV
Q-FCV-R	TGGGGCTRTCCATGTTGAT	
Q-IRF1-F	GGAAGTGAAGGACCAGAGC	qRT-PCR for detection of IRF1
Q-IRF1-R	TCCATCGGAGAAGGTATCA	
qIFITM1 F	CACCACCGTGATCAACATCCA	qRT-PCR for detection of IFITM1
qIFITM1 R	GACTTCACGGAGTAGGCAAAG	
qViperin F	CATGACCGGGGCGAGTACCTG	qRT-PCR for detection of Viperin
qViperin R	GCAAGGATGTCCAAATATTCACC	
qISG15 F	TCCTGGTGAGGAACCACAAGGG	qRT-PCR for detection of ISG15
qISG15 R	TTCAGCCAGAACAGGTCGTC	

## Data Availability

The data used to support the findings of this study are available from the corresponding author upon request.

## Supplementary Materials

Supplementary Materials
**Figure S1**: fe-IRF1 upregulates the mRNA expression of ISG15, IFITM1, and Viperin. CRFK cells were transfected with 500 ng/well of the pMyc-IRF1. At 24 h posttransfection, the mRNA expression of ISG, IFITM1, and Viperin was detected by qRT-PCR method. Error bars represent standard deviations and each sample was run in triplicate.*∗*: P<0.05; *∗∗*: P<0.01.
