# Supplementary material for: Identification of Feline Interferon Regulatory Factor 1 as an Efficient Antiviral Factor against the Replication of Feline Calicivirus and Other Feline Viruses
Source: Biomed Res Int. 2018 Jun 12;2018:2739830. doi: 10.1155/2018/2739830 (PMC6020669; doi:10.1155/2018/2739830)
Supplement: Supplementary Materials — Figure S1: fe-IRF1 upregulates the mRNA expression of ISG15, IFITM1, and Viperin. CRFK cells were transfected with 500 ng/well of the pMyc-IRF1. At 24 h posttransfection, the mRNA expression of ISG, IFITM1, and Viperin was detected by qRT-PCR method. Error bars represent standard deviations and each sample was run in triplicate.∗: P<0.05; ∗∗: P<0.01. [file 2739830.f1.pdf]

Figure. S1

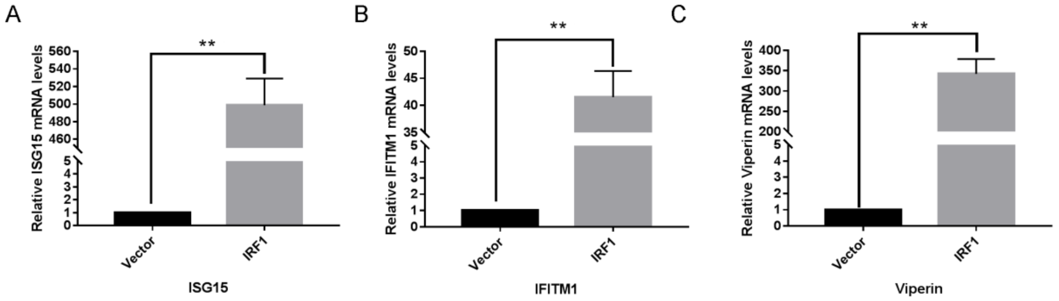

Figure S1. Fe-IRF1 upregulate the mRNA expression of ISG15, IFITM1 and Viperin. CRFK cells were transfected with 500 ng/well of the pMYC-IRF1. At 24 h post-transfection, the mRNA expression of ISG, IFITM1 and Viperin was detected by qRT-PCR method. Error bars represent standard deviations and each sample was run in triplicate.*, P<0.05; **, P<0.01.
